# The Diagnosis of Diseases of the Nervous System

**Published:** 1892-12

**Authors:** 


THE DIAGNOSIS OF DISEASES OF THE NERVOUS SYSTEM. A Manual for Students and Practitioners, by CHRISTIAN HERTER, M. D., Physician to the Class of Nervous Diseases, Presbyterian Hospital Dispensary. Pp. 628, 12mo, cloth. G. P. Putnam's Sons. New York and London: The Knickerbocker Press. 1892.

There is much to be said in favor of this book, for it is unusually clear and direct in style. It is fully abreast of modern thought and of the recently conducted experiments. The arrangement of the book is unusual. Instead of treating of diseases in rotation, he has adopted the plan of describing, first, methods of locating the seat of the lesion, beginning with the anterior portion of the brain and proceeding regularly to consider the symptoms which indicate lesions of the different cerebral areas. The cerebellum, Pons, medulla, and spinal cord are similarly considered. After a most admirable consideration of one means of locating, he then considers the method of diagnosticating the nature of the lesion. After this, the study of clinical types of disease is taken up. The chapter on The Differential Diagnosis of Functional and Organic Disease is one of the best in the book.

We do not hesitate in saying that it is one of the best books we have seen. It shows that it is the product of a well-ordered mind. J. W. P.

